# A multilevel evaluation of the development and implementation of a promotora-led nature-based intervention

**DOI:** 10.3389/fpubh.2026.1854138

**Published:** 2026-10-27

**Authors:** Shreya S. Aragula, Anne Saw, Jessica Rodriguez, Yenny Avila Vazquez, Viridiana Gonzalez, Monica Zohar, Catherine Game, Dani Abboud

**Affiliations:** 1 Chicago Asian American Psychology (CAAP) Lab, Department of Psychology, College of Science and Health, DePaul University, Chicago, IL, United States; 2 Academic Medical Center Department of Psychiatry, Mass General Brigham, Boston, MA, United States; 3 Department of Psychiatry, Harvard Medical School, Boston, MA, United States; 4 Health Equity and Nature (HEN) Accelerator, Brushwood Center Ryersonwoods, Riverwoods, IL, United States; 5 Highwood Library and Community Center (HLCC), Highwood, IL, United States; 6 Lotus Wellness Center, Palatine, IL, United States

**Keywords:** community health workers, community-based participatory research, health equity, implementation science, Latine mental health, nature-based interventions, third-wave cognitive behavioral therapy

## Abstract

**Background:**

Latine communities in the U. S. face persistent barriers to mental health care, exacerbated by inequities and trauma, requiring accessible and culturally congruent care. Simultaneously, these communities also possess profound strengths. To address these needs, Transforming Internal Experiences for Resilience and Restoration through Acceptance (TIERRA), an intervention led by Community Health Workers (CHWs, *promotoras* in Spanish) that combines third-wave cognitive behavioral therapy with Forest Bathing, was co-developed. The purpose of this paper is to detail the collaborative processes of TIERRA’s intervention development and implementation, and to report initial contextual evaluation outcomes.

**Methods:**

The subsequent pilot development and implementation was evaluated using the Practical Robust Implementation and Sustainability Model (PRISM) and the embedded Reach, Effectiveness, Adoption, Implementation, and Maintenance (RE-AIM) framework to assess capacity building, implementation, and preliminary outcomes.

**Results:**

TIERRA was developed utilizing Community-Based Participatory Research (CBPR) to center local community needs and assets. Its pilot demonstrated high feasibility and engagement (86% completion rate; *n* = 43). Evaluation of the implementation process revealed strong adoption and sustainability. Contextual evaluation with qualitative insights on effectiveness revealed the cultivation of radical hope, and that healing occurred through individual, interpersonal, community, and environmental pathways. Quantitative effectiveness outcomes showed significant reductions in stress (*p* = 0.026) and depression (*p* = 0.031), and significant increases in sense of community (*p* < 0.001) and sense of belongingness (*p* = 0.009).

**Discussion:**

TIERRA is a promising culturally relevant, CHW-led mental health intervention that promotes healing among Latine Americans. This study also shows that intervention development and implementation that centers community participation and attends to cultural contextual factors can build community capacity and is sustainable.

## 1 Introduction

Although Latine individuals constitute the largest minority population in the U. S., widespread gaps in health disparities persist between Latine Americans and non-Hispanic-White Americans (1). Latine adults experience significant mental health burdens, including elevated rates of depression, anxiety, and post-traumatic stress disorder (PTSD), yet they utilize formal mental health services at nearly half the rate of non-Hispanic White adults (2). The causes of such disparities are complex and multifaceted, stemming from the historical trauma of slavery and colonialism, forced displacement, and structural oppression (3). In addition, Latine immigrants also endure stressors pre-migration, during their journey into a host country, and after arriving (post-migration), carrying a cumulative burden that can result in the transmission of intergenerational trauma (4). These past experiences, coupled with the discrimination and racism encountered in the U. S., perpetuate a cycle of chronic stress, poverty, food insecurity, housing, and job-related concerns (5). These challenges are compounded by structural inequities within the built environment. A historical lack of sufficient public investment and urban planning that fails to account for historical trauma (e.g., redlining) restricts access to safe and restorative natural spaces, hindering neighborhood recovery (6, 7). Despite these compounding systemic burdens, Latine communities demonstrate profound cultural resilience, drawing on deep social connectedness, strong familial bonds, and shared agency to buffer against psychological distress (8). Yet, there remains a lack of evidence-based, culturally responsive efforts to promote positive mental well-being and healing for Latine communities (9). In this paper, we describe the community-based participatory research (CBPR) design and implementation of a culturally relevant and trauma-informed nature-based mental health-promoting intervention for a Latine community in Illinois. A glossary of relevant terms has been included in Table 1.

**Table 1:** Relevant terms.

Term/Framework	Category	Definition	Application within the TIERRA Intervention
TIERRA (Transforming internal experiences for resilience and restoration through acceptance)	Intervention model	A task-shifting, nature-based mental health intervention co-developed with and for the Latine community.	Serves as the 8-week core intervention curriculum combining forest bathing, and third-wave CBT skills, to address trauma and foster collective healing.
Community-based participatory research (CBPR)	Research methodology; the process of intervention development	A collaborative research orientation that equitably involves community partners, practitioners, and researchers in all phases of the study.	Guided the overall intervention development, method of co-design of intervention materials, and equitable promotora involvement and compensation.
Promotoras/Community health workers (CHWs)	Workforce/task-Shifting	Trusted frontline public health workers and community liaisons from the target population who share lived experiences, language, and cultural context with the participants they serve.	Served as trained co-facilitators and peer leaders delivering intervention sessions, supporting participant engagement, and navigating structural barriers.
Forest bathing/nature-based intervention (NBI)	A nature-based healing practice	Structured, sensory-based immersion, typically in natural outdoor settings designed to reduce physiological stress and promote mindfulness.	Provided the restorative component of the intervention, utilized guided sensory invitations (e.g., “befriending a tree,” sit spots, solo wanderings).
Radical hope	Psychological/healing framework	A collective psychological orientation that maintains faith and meaning-making toward alternative, thriving futures in the face of adversity. It is a component of radical healing.	The psychological component participants were working toward.
HEART framework (*Healing Ethno-Racial Trauma*)	Psychological/healing framework	A four-phase model (*Relief/Sanctuary, Grieving, Reconnecting, Resisting*) addressing ethno-racial trauma through cultural connection and collective processing.	Served as a framework that guided intervention components and cultural adaptation.
Trauma-informed care (TIC)	Clinical/organizational approach	A framework recognizing the impact of trauma, emphasizing physical, psychological, and emotional safety for both providers and participants.	Informed inviting language and environment, and manual adaptations
Third-wave cognitive behavioral therapy (CBT)	Clinical/psychotherapeutic practice: an evidence-based psychotherapy approach	Modern psychotherapeutic approaches (e.g., ACT, DBT) emphasizing mindfulness, context, emotional acceptance, and values-guided behavioral change.	Integrated via skill-building activities primarily in Dialectical Behavior Therapy and Acceptance and Commitment Therapy.
Dialectical behavior therapy (DBT)	Clinical/psychotherapeutic practice: a type of third-wave psychotherapy that is focused on mindfulness.	A specific therapy aimed at teaching skills based on the practice of mindfulness. Behavioral strategies aimed at tolerating, accepting, and surviving painful emotions or acute crises without resorting to unhelpful reactions.	Adapted to serve the Latine community; components were integrated with forest bathing practices
Acceptance and commitment therapy (ACT)	Clinical/psychotherapeutic practice: a type of third-wave psychotherapy that is focused on mindfulness.	A behavioral therapy focusing on psychological flexibility, mindfulness, and cognitive defusion (“unhooking” from difficult internal states).	Adapted to serve the Latine community; components were integrated with forest bathing practices
Post-traumatic stress disorder (PTSD)/	Diagnostic/clinical term	Psychological distress resulting from directly experiencing, witnessing, or learning about severe, life-threatening, or systemic traumatic events.	Measured by the PCL-5 Scale
Depression	Diagnostic/clinical term	Psychological distress characterized by persistent low mood, loss of interest or pleasure, fatigue, cognitive difficulties, and vegetative symptoms that impair daily functioning.	Measured by the PHQ-9 Scale
Anxiety	Diagnostic/clinical term	Excessive, uncontrollable apprehension and worry accompanied by heightened autonomic arousal, somatic tension, and persistent hypervigilance.	Measured by the GAD-7 Scale
Perceived Stress	Psychosocial/health psychology construct	The degree to which life situations are appraised as unpredictable, uncontrollable, and exceeding an individual’s perceived resources and capacity to cope.	Measured by the Perceived Stress Scale (PSS)
Sense of community	Psychosocial/community psychology construct	The subjective perception of belonging, mutual interdependence, shared emotional connection, and collective influence within a defined social or geographic group.	Measured by the Brief Sense of Community Scale (BSCS)
General sense of belonging	Psychosocial/relational well-being construct	The fundamental psychological experience of feeling accepted, valued, and integrated within one’s broader social environment and relationships.	Measured by the General Sense of Belonging (GSB) Scale
PRISM (Practical robust implementation and sustainability model)	Implementation science framework	An implementation model that identifies broad multi-level contextual factors (external environment, organizational climate, recipient characteristics) influencing program success.	Provided the broader contextual evaluation lens to assess community fit, infrastructure barriers (e.g., transit, childcare), and sustained adoption.
RE-AIM (Reach, effectiveness, adoption, implementation, maintenance)	Implementation science framework	An evaluation framework designed to systematically measure the public health impact, fidelity, and translatability of health interventions.	Structured the reporting of implementation outcomes, including participant reach, completion rates, attendance fidelity, and multilevel effectiveness.

Following the Ecological Validity Framework, mental health interventions must address language, metaphors, methods, and delivery contexts to ensure cultural resonance (10, 65). Interventions should also incorporate observable cultural characteristics such as music, food, location, places, and people, as well as deeper psychological, historical, environmental, and socio-cultural contexts (11). Intervention development is most successful when using Community-Based Participatory Research (CBPR) to center a population’s lived experiences, agency, and cultural conceptualizations of healing (10). For Latine communities, group-based interventions led by *promotoras* (community health workers) offer less-stigmatizing alternatives to clinical settings (12). Incorporating Trauma-Informed Care (TIC) allows these models to address the layered traumas experienced by Latines (13). Situating these promotora-led interventions in nature offers a promising contextual adaptation; natural environments can provide a culturally relevant, restorative environment that facilitates physiological and psychological healing (14–17).

For the Latine community specifically, the Healing Ethno-Racial Trauma (HEART) framework can inform culturally tailored, trauma-informed intervention development. This framework centers the distress resulting from exposure to racism, discrimination, and structural oppression (i.e., ethnoracial trauma) while identifying inherent cultural strengths, such as determination, *esperanza* or hope, adaptability, a strong work ethic, connectedness to others, collective emotional expression, and a desire for resistance (8). HEART posits holistic healing, i.e., recovery that occurs across individual, family, and community levels through establishing *sanctuary spaces* (defined as non-judgmental environments that promote psychological safety). The model also facilitates collective grieving, encourages reconnection with cultural roots, and promotes engagement in resistance (8).

Building upon the HEART framework, radical or *collective* hope seeks to equip marginalized communities with the agency to pursue a more egalitarian future amid ongoing oppression (18, 19). Conceptually, radical hope shares an emphasis on culturally grounded healing, collective resistance, and collective resilience with HEART (8, 19). Mosley et al. (20) state that cultivating radical hope requires acknowledging oppression, understanding past resistance, practicing ancestral pride, envisioning an egalitarian world, and actively pursuing social justice. Specifically, embodying radical hope requires an orientation toward both the individual and the collective, seamlessly interweaving the past and future through the threads of collective memory, faith, and agency (19). While research exploring the direct link between radical hope and psychological outcomes is still in its infancy, this framework may provide a vital link for contextualizing and supporting interventions for Latine communities, connecting individual outcomes to collective and systemic change.

Evidence suggests that trauma-informed mindfulness-based interventions (MBIs), including third-wave cognitive behavioral therapies like dialectical behavior therapy and acceptance and commitment therapy, are effective for Latine and other POC communities (20–22); yet, despite their roots in Eastern philosophy, these interventions were primarily developed for White, affluent, and highly educated populations (23). Moreover, despite efforts to expand reach, current MBI dissemination models remain fraught with barriers, including limited language options, inaccessibility of locations for lower-SES individuals, and a lack of culturally relevant stories and metaphors (24, 25). These barriers alienate diverse communities that might otherwise find common ground between mindfulness and their own contemplative or spiritual practices (26, 27).

Forest bathing and other less formal mindfulness Nature-Based Interventions (NBIs) offer an alternative for POC communities by countering the distrust often associated with more clinical settings (15). Derived from *Shinrin-Yoku*, forest bathing is a restorative, trauma-informed practice that involves slowing down and immersing oneself in a natural environment to mindfully engage one’s senses (27). In addition to its philosophical and spiritual alignment with mindfulness, evidence suggests that forest bathing helps improve cardiovascular health as well as mental well-being (27, 28). Despite this potential, forest bathing has yet to be culturally adapted for U. S. communities. Yet, the congruence between MBIs and NBIs (including forest bathing) lends itself to an integrated approach: a model especially relevant for Latine and other POC communities with deep ancestral and Indigenous ties to nature and land-based healing practices.

Given the complex historical and contemporary traumas faced by Latine and other POC communities, developing culturally resonant interventions requires centering community voices and agency. Community-Based Participatory Research (CBPR) approaches achieve this by actively involving the community in adapting and developing interventions, ensuring relevance to local and cultural contexts while facilitating co-learning, capacity-building, and sustainability (11). To complement this active development process, leveraging an implementation science framework helps mitigate the tension between scientific rigor and the flexibility necessary for cultural tailoring by allowing researchers to systematically document these community-driven adaptations.

Moreover, while symptom reduction is a critical metric of success, traditional interventions may not achieve sustainability in marginalized communities because of a lack of focus on the impact of the intervention on local environmental and organizational contexts. A core tenet of CBPR is capacity building, i.e., an intentional effort to strengthen the skills, infrastructure, and resources of a community. Therefore, its evaluation must include individual, systemic, and contextual changes.

Seeking to bridge the gap between traditional psychotherapy, current limitations in MBIs and NBIs, and the unique needs of marginalized populations including Latine communities, in this paper, we introduce Transforming Internal Experiences for Resilience and Restoration through Acceptance (TIERRA). TIERRA, Spanish for “land/earth,” is a culturally grounded intervention integrating third-wave CBT with forest bathing that was co-developed for and with the community using CBPR principles. To capture how this community-engaged approach builds local capacity and supports sustainability, the specific aims of this paper are twofold: first, to detail the contextual, CBPR-driven development process of TIERRA; and second, to evaluate its multi-level implementation outcomes utilizing an implementation science framework.

## 2 Methods

### 2.1 Study context

In July 2022, the neighboring communities of Highwood and Highland Park, Illinois experienced a mass shooting that left residents fearful, grieving, and deeply distressed (29). Despite being separated by less than five miles, these communities are divided by pronounced structural disparities. Highwood, with a Hispanic population of nearly 40%, has faced structural challenges, including gentrification-fueled property inflation with little to no corresponding improvement in socioeconomic status. Compared to Highland Park, Highwood reports significantly higher rates of uninsured individuals, unemployment, and disability qualification. Moreover, Highwood’s poverty rate is at least four times higher than that of Highland Park (30). The shooting incident served as an acute traumatic stressor that layered onto these existing chronic structural challenges. This urgent need for restoration led the Highwood Library & Community Center (HLCC), a community organization in the neighborhood, to seek trauma-informed and culturally sensitive mental health services for its Latine community.

Simultaneously, the Brushwood Center at Ryersonwoods, Lake County, Illinois, a not-for-profit focused on nature, arts, and health equity, collaborated with the Chicago Asian American Psychology (CAAP) Lab at DePaul University for a needs assessment in Lake County, Illinois. The assessment, which focused on experiences of nature access and health, showed that, despite ample green and blue spaces in the county, access to and utilization of these spaces was limited to individuals from higher socio-economic statuses. However, for those who could access it, nature was found to provide multiple mental health benefits. Findings also suggested that nature was viewed as a safe, nonjudgmental, and nondiscriminatory space. Participants reported that outdoor experiences were perceived as especially meaningful when individuals spent time outside with loved ones, and that accepting nature for both its protective and destructive properties encouraged acceptance of the full range of emotions and life experiences that participants had endured (31). Importantly, these conditions mirror experiences of mindfulness and grounding, and are considered imperative components of trauma-informed care; these results suggest that nature can provide a safe space for healing from internal pain (27, 32, 33).

#### 2.1.1 Community partnerships

Building upon a long-standing partnership, the HLCC, and the Brushwood Center recognized a unique opportunity to develop a nature-based mental health intervention. During the pandemic, the HLCC had successfully launched promotora-led nature walks to encourage vaccinations, stress relief, and a safe return to outdoor spaces, following the mass shooting. The success of this program, combined with the CAAP Lab and Brushwood’s findings on nature and health equity, suggested potential for a more robust mental health intervention. By leveraging Lake County’s ample natural spaces (community assets) and HLCC’s capacity and assets—including its promotoras—the researchers at CAAP Lab, Brushwood, and HLCC staff co-developed TIERRA, a promotora-led nature-based mental health intervention.

### 2.2 Positionality statement

The authors of this paper include individuals from diverse lineages, identities, sexualities, genders, and lived experiences, bound by a shared commitment to equity and the restorative power of community and nature. We are composed of non-citizens, immigrants, and children of immigrants, bridging perspectives from the global south and the local community. Despite a lack of shared linguistic and cultural heritage, one author (SSA), a doctoral candidate in clinical-community psychology and an international student from India, shares common experiences of immigration and belongingness in a new land with the promotoras and Latine residents of Highwood. Our work is guided by the knowledge and experience of an associate professor of clinical-community psychology (AS) who identifies as Asian and has a family history of forced migration. It is enhanced by a co-author (JR), a certified forest bathing guide with a primary degree in Sociology and Environmental Studies. As a Latine-identifying individual from Lake County, they ensure that TIERRA remains an authentic reflection of the community it serves. Similarly, the cultural relevance was bolstered by three Bilingual trainers (YA, VG, and MZ) representing HLCC, who also share culture, cultural identities, language, location, and a history of immigration with the promotoras and community participants. Enriched by the institutional wisdom of Brushwood Center (DA and CG), TIERRA represents our shared effort to dismantle structural racism and bridge the historical lack of access to nature and healing for underserved communities.

### 2.3 Study design

A community-engaged, mixed-methods framework was used. CBPR served as the overarching methodology for intervention development (11, 34). To systematically evaluate both the capacity built during the development and success of the intervention, the study employed an implementation science approach, guided by the Practical Robust Implementation and Sustainability Model (PRISM) (33, 34). PRISM was used to assess contextual and structural shifts because of the CBPR process, while its embedded RE-AIM dimensions (Reach, Effectiveness, Adoption, Implementation, and Maintenance) guided the mixed methods evaluation of the pilot’s impact and feasibility (35).

### 2.4 Implementation science frameworks

PRISM provides a lens to systematically capture the multi-level factors shaping the development and subsequent outcomes of this intervention (34). It contextualizes outcomes across four domains: (1) perspective on the program or intervention design, (2) the external environment, (3) the implementation and sustainability infrastructure, and (4) multi-level recipient characteristics (34), also highlighting system-level interactions within and outside the intervention (33).

PRISM also embeds Reach, Effectiveness, Adoption, Implementation, Maintenance (RE-AIM) outcomes within its framework to evaluate the impact of the intervention. RE-AIM outcomes are viewed as interdependent, resulting in an iterative process of feedback, tailoring, and intervention adaptation (34–36). The application of CBPR methodology alongside PRISM (and its embedded RE-AIM) is not just complementary but highly recommended (37). By centering the needs of marginalized communities, this integration can propel intervention research toward health equity while ensuring rigorous implementation.

### 2.5 Participants

The bilingual trainers and promotoras, who co-developed the intervention, were recruited by word-of-mouth based on a community partnership between the Brushwood Center and the HLCC. Community participants were initially recruited through a listserv generated by the HLCC or by word-of-mouth by the promotoras, who spread word of the intervention while they were working at other HLCC events. A script was followed for recruitment. These recruitment methods were chosen to improve trust and reduce stigma for the community (37). While sample size calculations for pilot studies are not always deemed necessary and are typically dependent on the goal of the intervention, a sample size of 30–60 individuals is commonly targeted (38, 66). A power analysis was conducted using G*Power Version 3.1.9.7 (39, 40, 67). For a two-tailed paired-samples t-test with an alpha level of 0.05 and a power of 0.80, it was determined that a sample size of 34 participants would be sufficient to detect a medium effect size (*d* = 0.50). This effect size is consistent with those typically observed in evidence-based psychotherapy interventions for similar populations (39, 40). Therefore, after consulting with the HLCC and considering potential attrition rates, a goal of reaching 50 Latine-identifying adults was targeted. Inclusion criteria for the community intervention included: (1) self-identifying as Latine; (2) being 18 years of age or older; and (3) currently residing in and around Highwood, Illinois.

Overall, three bilingual trainers, five promotoras, and 47 community members provided insights for the pilot intervention. All participants identified as Latine and as community members affiliated with the HLCC. Among the bilingual trainers, the mean age was 38.67 (SD = 7.37), ranging from 33 to 47. All three identified as female (100%); one trainer identified as Latinx/Mexican (33.3%) and two as Latine (66.6%). Two were practicing clinicians (66.6%), and one was a case manager (33.3%). The promotoras’ mean age was 40.0 (SD = 6.52), ranging from 29 to 46. All five were identified as female (100%). One promotora identified as Latino/Hispano (20%); one as Latina Mexicana (20%); two as Mestiza (40%); and one as Mestizo (20%). The final community sample consisted of participants with a mean age of 47.5 years (SD = 14.4), ranging from 18 to 79. Most of the sample identified as female 88.8%, while 5.9% identified as male, and 5.8% did not specify their gender. In terms of ethnicity, 43.14% identified as Latinx or Hispanic, 15.69% as Mestizo, 9.8% as Mexican/Mexicana, and 31.37% picked “Other” or no response. Other details about participant demographics have been omitted for privacy.

### 2.6 Procedure

The community intervention was conducted in Lake County, Illinois between April 2024 and June 2025. The procedure followed a multi-phased, mixed methods design grounded in CBPR principles and operationalized through the PRISM framework (33, 34). The phases included: (1) a train-the-trainer phase, wherein intervention developers trained bilingual therapists and a case worker on the intervention over 3 days; (2) Promotora training, wherein the trainers trained promotoras with continued support from intervention developers over 8 weeks (a total of 40 h); and (3) Community intervention, wherein promotoras conducted the intervention in the community with ongoing support from bilingual trainers and intervention developers. The 40-h promotora training covered vicarious trauma, ethical boundary setting, and included a thorough walk-through of all sessions paired with the manual. Promotoras also engaged in role-playing exercises.

During recruitment, interested individuals contacted the research team via email. Eligibility and interest in post-intervention interviews were assessed through a screening questionnaire administered via Qualtrics. Eligible participants received an electronic informed consent document; once consent was obtained, participants completed a baseline survey assessing psychological well-being and demographics (e.g., age, gender, race/ethnicity, residency status, income; optional to protect participant privacy). The intervention lasted approximately 8 weeks. For ongoing support, the promotoras, bilingual trainers, and research team met weekly to debrief sessions, and the promotoras and trainers completed reflection surveys after each session. Upon completion of the intervention, community participants completed a post-intervention survey measuring the same psychological constructs as the pre-survey. Following the intervention, qualitative data were collected in the form of a focus group with the bilingual trainers, a focus group with the promotoras, semi-structured, 30-min individual interviews with consenting community participants, and a 30-min interview with the translator of the manual and measures. The study protocol was approved by the Institutional Review Board (IRB) of DePaul University. Additional details regarding the procedure are included in the results section of this paper.

### 2.7 Intervention

TIERRA is an 8-week group curriculum meeting weekly for 2 h. Delivered in Spanish by promotoras, the program is grounded in the HEART and Radical Hope frameworks and integrates third-wave CBT with Forest Bathing (8, 18, 19, 25, 27, 41, 42). The primary goals of TIERRA are to help participants identify stress, foster meaningful relationships with nature and community, and increase their sense of belonging. Accordingly, sessions focused on evidence-based skill-building in mindfulness, grounding, distress tolerance, and emotional regulation.

Weather permitting, sessions took place at the Openlands Lakeshore Preserve at Fort Sheridan, Illinois (approximately one mile away from the HLCC). This conservation area included green and blue space, including unpaved trails, and ravines, along Lake Michigan. On colder days, or during periods of rain, sessions took place at a neighborhood park opposite the HLCC, and at the HLCC itself, which served as a walkable, and easily accessible sanctuary space. Irrespective of where the intervention occurred, participants met initially at the HLCC to utilize childcare services and coordinate carpools to the preserve when necessary.

To maintain predictability, the format for a typical 2- h session included (i) an arrival and welcome (5 min), (ii) a forest bathing or mindfulness exercise (30–50 min); (iii) skills-building or psychoeducation (30–50 min), and (iv) a closing tea circle (15 min). Following each group meeting, participants engaged in independent at-home practices directly related to that week’s session content (see Supplementary Table A1 for a description of sessions).

### 2.8 Metrics for evaluating results

#### 2.8.1 Co-development using CBPR and PRISM

Evaluation metrics surrounding development were guided by CBPR principles and subsequently organized into the PRISM framework (11, 34). Development prioritized core CBPR principles such as building on community strengths rather than solely focusing on deficits or needs; fostering collaboration, equity, and capacity building; and emphasizing local relevance and ecological perspectives through a reflexive and iterative process (43, 44). These principles connect to the following PRISM domains: (1) program or intervention design, considering how the intervention or program is patient-centered, addressing barriers to care, and estimating impact and use; (2) the external environment, including policies and local resources, (3) the implementation and sustainability infrastructure, including training support, feedback mechanisms, and resources, and (4) multi-level partner characteristics, including organizational characteristics to support behavior change and patient characteristics (34).

#### 2.8.2 Measuring implementation outcomes

Implementation outcomes included PRISM contextual domains, including embedded RE-AIM metrics (34, 35). Data were collected during training, before the community intervention (pre-intervention data), and after the community intervention (post-intervention data). Outcomes were measured qualitatively and quantitatively. Qualitative data were gathered through weekly reflection surveys completed by the bilingual trainers, promotoras, and the research team during the promotora training and the community intervention; semi-structured focus groups with the promotoras and bilingual trainers after the intervention; and semi-structured individual interviews with participants. All participants were given the option to provide qualitative data in English or Spanish. Promotoras completed reflection surveys and participated in focus groups in Spanish. Quantitative data regarding promotora and participant attrition, session attendance, participant distress measured via the Perceived Stress Scale [PSS-10; Cohen et al. (45)], the Patient Health Questionnaire [PHQ-9; Kroenke et al. (46)], the Generalized Anxiety Disorder scale [GAD-7; Spitzer et al. (47)], and the PTSD Checklist for DSM-5 [PCL-5; Weathers et al. (48)], and belongingness (measured via the General Sense of Belongingness Scale [GBS; Malone et al. (49)] and the Brief Sense of Community Scale [BSCS; Peterson et al. (50)] were collected via pre- and post-intervention surveys. Participants were given the option to complete these surveys in either English or Spanish.

## 3 Results

Results are organized by the development process, followed by the intervention outcomes. We first describe the CBPR process of developing TIERRA, organized by PRISM dimensions. Table 1 shows the implementation of CBPR for intervention development, while Table 2 summarizes intervention development along PRISM dimensions. We then present pilot evaluation outcomes, assessed through the PRISM and embedded RE-AIM framework, which are further summarized in Table 3.

**Table 2:** CBPR best practices and TIERRA implementation.

CBPR best practice	TIERRA application
Recognize community as a unit of identity	Defined the community through a shared Latine identity in Highwood and Lake County, focusing on common linguistic, cultural, and historical experiences.
Build on community strengths and assets	Shifted from a deficit only model to an asset-based approach by utilizing the Latine community’s cultural strengths (e.g., esperanza) and leveraging local “green and blue” spaces as “healing sanctuaries,” as outlined by the HEART framework. CHWs were also identified as community strengths.
Equitable collaboration in all research phases	Equitably involved diverse partners—including *promotoras*, community members, and local centers—in every stage from conceptualization and design to implementation and dissemination.
Equitable dissemination of results	Delivering results in multiple formats – a community report for community stakeholders and interested participants, a more systemic report for funders, posters and publications for researchers and academics, an art exhibition consisting of participant interpretations of the intervention for community participants and their families
Promote Co-learning	Promotoras advocated for cultural needs and nuances (focus on adaptation), while bilingual trainers and researchers supported intervention structure (fidelity)
Capacity building	Used a CHW-model for dissemination of the intervention. Also adopted a train-the-trainer model where trainers trained *promotoras*. Trainers and *promotoras* were local community members.
Critical insiders	Integrated “critical insiders” (a Latine forest bathing guide and *promotoras*) who used their positionality and established networks to identify essential curriculum components like nature integration and distress tolerance.
Emphasis on local relevance and ecological perspectives	Shaped the intervention design to address the specific socio-political stressors of Lake County and the acute trauma of the July 2022 Highland Park mass shooting, while using trusted sites like the HLCC.
Reflexive and iterative development process	Established a Community Advisory Board (CAB) for iterative feedback and collected weekly reflection surveys to refine the program in real-time based on the dual needs of providers and community members.
Commitment to long-term sustainability	Secured multi-year grant funding and developed modules to tackle compassion fatigue, ensuring the longevity of the delivery staff and the community’s capacity to maintain the program. CHW-model as sustaining the intervention in the community.

**Table 3:** CBPR methodology mapped onto PRISM contextual considerations.

PRISM domain	TIERRA utilization
Intervention design	Co-developed using CBPR principles, the HEART framework, and Radical Hope. “Critical Insiders” and a Community Advisory Board ensured the integration of third-wave CBT and forest bathing was culturally and linguistically adapted to address structural inequities.
Recipients (Participants)	Targeted Spanish-speaking Latine adults in Lake County, IL. Leveraged community strengths like cultural resilience and *familismo* to address cumulative distress, health inequities, and local idioms of distress.
Recipients (Facilitators)	Shifted from a purely therapist-led model to a community-informed, *promotora*-led task-shifting model. Addressed their dual role as community members and frontline workers by integrating training on vicarious trauma to increase trust, reduce burnout, and ensure scalability.
External environment	Navigated systemic barriers to care by intentionally utilizing free-entry local green and blue spaces. Directly addressed cumulative distress stemming from systemic racism, pandemic isolation, and the acute community trauma of the July 2022 Highland Park shooting.
Implementation and sustainability infrastructure	Utilized a “Train-the-Trainer” model and existing partnerships with local organizations (Highwood HLCC, Brushwood Center) to build workforce capacity. Established a pathway for maintenance through tiered dissemination and institutionalized programming.

### 3.1 Co-development using CBPR and PRISM

#### 3.1.1 The external environment

Guided by the PRISM framework and utilizing CBPR principles, the intervention was designed to consider the external environment, including local assets, local structural constraints, and contextual factors impacting the local community at that time. Given the presence of ample green and blue spaces in Lake County, these community assets became the vital locations for the intervention. To make these spaces accessible to the Latine community, special efforts were made to ensure that the green and blue spaces chosen had free entry and were relatively easy to access. This use of natural space was intentional and aimed at reducing identified structural and nature access inequities. Since TIERRA was conceptualized shortly after the pandemic, when social distancing was being practiced, there was an increase in people spending time outdoors for stress relief. Further, the intervention design was specifically shaped by the acute trauma of the July 2022 Highland Park mass shooting and the ongoing socio-political stressors affecting Lake County’s Latine population. By utilizing a CBPR asset-based approach, TIERRA turned the HLCC—a trusted community organization—into the primary site and source for implementation, bypassing the structural and psychological barriers associated with traditional clinical settings.

#### 3.1.2 Perspectives on the intervention design

Special efforts were made to integrate the perspectives of multiple stakeholders involved in the project. Specifically, consistent with CBPR principles, this project equitably involved community partners (promotoras, Lake County and Highwood Community members, the Brushwood Center, HLCC, DePaul University, and other experts) in all stages of the research process—from identifying needs and assets to intervention design, implementation, and dissemination. Multiple meetings with Brushwood, HLCC, and the promotoras allowed the research team to identify key community resources, strengths, needs, and priorities.

##### 3.1.2.1 Critical insiders

The clinical and contextual components of the intervention were further enhanced by the perspective of a partial insider, i.e., certified forest bathing guide (JR) from Lake County, who identifies as Latine, joining the research team to develop the nature-based healing components. Furthermore, a community advisory board, consisting of Latine-identifying Highwood and Lake County individuals, Latine-identifying promotoras unaffiliated with Highwood, but working in Lake County, two forest bathing guides, one therapist, and three bilingual researchers in the fields of community and clinical psychology, was established to receive iterative feedback throughout the intervention development and implementation process. The perspective of the translator, who was bilingual and a therapist, was obtained as well.

As “critical insiders,” the promotoras used their unique positionality and established community networks to shape the intervention. Based on their consistent dialog with community members, they identified essential intervention components, including nature integration, need for an emphasis on values, acceptance, distress tolerance, and interpersonal effectiveness. Their prior training and comfort were also key considerations: since they had received training in the HEART framework and its relevance to the population at hand, it was integrated into the curriculum (8). Furthermore, upon their request, their training also included ways to tackle compassion fatigue and vicarious trauma, ensuring that the program remained grounded in the specific sociocultural context and the dual needs of the promotoras as both community representatives and frontline healthcare providers. In addition, the researchers received feedback from promotoras weekly during training through promotoras’ reflection surveys and a post-intervention focus group.

##### 3.1.2.2 Co-development of intervention content

Through an extensive literature review and collaborative meetings between the research team and the certified forest bathing guide (JR), a curriculum that integrates third-wave CBT with forest bathing was developed. These practices, along with their respective origins and cultural histories, were discussed and combined in a manner relevant to collective cultures and the Latine community. Specifically, each session paired mindfulness-based psychoeducation with a complementary, nature-based experiential exercise and included a discussion about the cultural relevance of the session topic. More information about session content is available in Supplementary Table A1. For instance, when covering the module on interpersonal effectiveness, psychoeducation centered on self-advocacy, communicating with loved ones, cultural modes of communication, and a discussion of the impact of *machismo*. The complementary forest bathing invitation prompted participants to venture into the forest to gather natural “gifts” for other group members. Similarly, when introducing dialectical behavior therapy skills for emotion regulation, facilitators adapted the psychoeducation to be more discussion-based. Acknowledging the structural inequities that often limit adequate sleep and healthy eating, the curriculum invited participants to reflect on how to make restorative choices within their current capacity. The corresponding forest bathing exercise involved grounding using a stone.

To ensure cultural congruence, all intervention materials, including the manual, participant workbook, and training presentations, were carefully adapted. Metaphors, narratives, and language were tailored to reflect the lived experiences of the local Latine community, anchored in the HEART framework and principles of radical hope. The HEART framework influenced the intervention group’s format through its inclusion of discussions about trauma and social justice, the promotion of nature as a “sanctuary space,” and the conceptualization of healing beyond an individual level. Concurrently, each psychoeducational module targeted a distinct pathway of radical hope, cumulatively building toward its full realization by the intervention’s conclusion. To help participants experience this growth, a “wishing tree” exercise was utilized. In the initial session, participants externalized individual and communal worries by placing them on the tree and rating their impact on their well-being. During the final session, participants revisited the tree to reflect on how their relationship to these stressors had shifted. The exercise culminated with participants writing down their hopes and dreams for themselves and their community.

The process of cultural adaptation involved multiple steps – first, materials were translated by a bilingual therapist, who was not associated with Highwood (VA). Subsequently, materials were further screened and adapted by the bilingual trainers at Highwood, bilingual members of the larger research team, and the CAB, which included local community members. Additionally, during training, iterative feedback obtained from the promotoras through reflection papers and weekly meetings was integrated into the program materials before the delivery of the community intervention. Cumulatively, the lived experiences and wisdom of the CAB, promotoras, bilingual trainers, and the translator aided in ensuring cultural, linguistic, and contextual relevance in the materials and in decreasing power imbalances. Their inclusion also helped in ensuring that various community members were collaborating on meaningful work for the community (51).

#### 3.1.3 Multi-level recipient characteristics, sustainability, and implementation structure

As mentioned above, the demographic, cultural, and contextual characteristics of the participants, individuals delivering the intervention (promotoras), as well as research and program staff across organizations, were central to our intervention design. Our reliance on community strengths and pre-existing community partnerships facilitated communication about project expectations and organizational values, with all organizations converging on the goal of empowering this underserved community and reducing health inequities. Organizations involved, namely the Brushwood Center and the HLCC, were grant-funded for a period of 3 years for projects about health equity. Funding from these grants helped facilitate intervention development and sustainability.

The CHW-led model was vital to the implementation infrastructure of the project. To train the promotoras and bridge linguistic differences, a train-the-trainer model was adopted. This method is often used in health care and nursing, and is found to have favorable outcomes (49, 68). It assisted in building local capacity, sustainability, and community empowerment, given that the bilingual trainers also lived in Lake County and the promotoras had a built-in, culturally attuned support system. During a three-day intensive training, the research team lead (SA) and forest bathing guide (JR) trained three Latine-identifying, bilingual trainers (MZ, VG, YA) affiliated with the HLCC on intervention components and delivery. The bilingual trainers then trained the promotoras during weekly meetings over the course of 2 months, while the research team helped and supported the endeavor. The promotoras then conducted the intervention over 8 weeks. During the community intervention phase, the promotoras met with the bilingual trainers and research team weekly for additional support. Upon completing data analysis, in accordance with CBPR principles, a tiered format was utilized for the dissemination of study findings to ensure accessibility and relevance across diverse audiences. For community participants, a community-focused report was developed with a primary emphasis on intervention results. For stakeholders and funders, a more comprehensive report was provided, which detailed the methodological frameworks in addition to the empirical results. To communicate results with the local community, a zine will also be created. Additionally, all community stakeholders were invited to participate in an art exhibition held at the Brushwood Center, featuring artistic representations of participants’ time together in the intervention.

Table 2 provides more information about the different ways in which CBPR was used, while Table 3 provides more information on how PRISM was conceptualized.

### 3.2 Implementation outcomes

To evaluate the intervention, RE-AIM outcomes were used, along with PRISM for contextualizing domains (Table 4).

**Table 4:** TIERRA implementation and PRISM/RE-AIM factors.

RE-AIM domain	PRISM contextual level	Evaluated indicators and contextual factors	Pilot implementation and effectiveness outcomes
Reach (Absolute number, proportion, and representativeness)	Individual, community, and external environment	Target enrollment vs. actual. Program completion rates Average session attendance per participant *Contextual Factors:* Cumulative distress, structural inequities, and acute trauma (e.g., July 2022 Highland Park shooting).	*Enrollment*: Target = 50 Latine individuals from Highwood; Actual registered = 46. *Completion*: *N* = 43 completed the program (86% of the target goal). Attendance: 81% *Barriers and adaptations*: Primary barriers were work conflicts and childcare (specifically transportation). To mitigate this, supervised childcare was provided at the HLCC, significantly bolstering retention. Using the library as an asset bypassed structural/psychological barriers of clinical settings.
Effectiveness (Impact on clinical, community, and qualitative outcomes)	Individual, community, and intervention design	Reduction in clinical symptoms. Growth in community belongingness *Design context:* Grounded in the HEART framework and Radical Hope.	*Clinical (Quantitative)*: Significant reductions in depression (*d* = −0.32, *p* = 0.031) and stress (*d* = −0.34, *p* = 0.026). Not significant for anxiety (*p* = 0.45) or PTSD (*p* = 0.48). For more information on these results, please refer to Supplementary Table A2. *Community (Quantitative)*: Significant increases in sense of community (*d* = 0.56, *p* < 0.001) and general sense of belonging (*d* = 0.38, *p* = 0.009). For more information on these results, please refer to Supplementary Table A2. *Qualitative*: Healing occurred across four pathways (individual, interpersonal, community, environment). Radical hope was conceptualized as practicing presence, allowing for dialectics, and accepting the past while striving for a better future. A more mindful and reciprocal relationship with nature was established. Stressors such as parenting, immigration and current socio-political climate were identified, with the intervention serving as a buffer.
Adoption (Proportion of settings and intervention agents)	Managers (Trainers), Staff (*Promotoras*), and Organizations	Proportion of eligible staff trained. *Contextual Infrastructure:* Utilized a CBPR “Train-the-Trainer” model to build local, culturally attuned capacity.	Managers (Trainers): 100% (3 of 3) of HLCC bilingual trainers received research team training and subsequently trained the *promotoras*. Two attended all 10 sessions; one missed 3 sessions due to work. Agents (*Promotoras*): 100% (5 of 5) received training/certification. Due to one role restructuring, 80% (4 of 5) completed the community delivery. Settings: Secured active partnerships (HLCC, Brushwood, DePaul) leveraging local assets (free-entry green spaces).
Implementation (Fidelity to protocol and cultural adaptations)	Staff (*Promotoras*), intervention design, and external environment	Fidelity to core concepts and staff satisfaction. *Contextual adaptations:* Iterative feedback from “Critical Insiders” (Latine forest bathing guide, CAB).	Acceptability and fidelity: Despite a steep learning curve, *promotoras* reported high satisfaction; minimal changes were made to core concepts. Training actively addressed vicarious trauma to reduce burnout. Cultural adaptations: CAB helped adapt metaphors and concepts (e.g., modifying DBT “PLEASE” skills to reflect structural barriers to sleep/diet). Logistical/external adaptations: A delayed start pushed sessions into colder months, requiring indoor plant replication for two sessions. The 2024 election delayed sessions by 1 week.
Maintenance (Institutionalization of the intervention)	Individual, staff, and organizations	Individual desire for ongoing support. Future recruitment initiatives Staff intent to continue delivery. *Contextual infrastructure*: Multi-tiered dissemination to sustain community buy-in.	Individual: Participants described the impact of the intervention to friends and community members, resulting in a waitlist for the next round of interventions; and little to no organizational efforts for recruitment for upcoming groups Individual: Participants requested a recurring support group for those who already completed the intervention; Brushwood Center responded by developing a monthly “Radical Hope and nature” art group. (1-year longitudinal survey planned). Staff: 2 of the 5 trained *promotoras* plan to deliver 3 interventions in Summer 2026. Future maintenance will track 2-year recertification. Organizational: HLCC committed to institutionalizing TIERRA, offering it at least once annually. Dissemination includes stakeholder reports, community zines, and a participant art exhibition.

## 4 Discussion

The current study elucidates the development and initial implementation of TIERRA, a novel community health worker-led nature intervention designed to promote nature access and mental health for Latine communities. It highlights the use of CBPR practices and attention to context, culture, and capacity building to support sustainability. The development of TIERRA utilized CBPR best practices, including co-learning between interventionists and community members serving as critical insiders, equitable collaboration and shared decision-making, and iterative and reflexive processes. As a promotora-led, nature-based group intervention, TIERRA tackles both acute traumatic stressors—such as the 2022 Highland Park mass shooting—and the chronic, cumulative burden of intergenerational and structural trauma through its integration of mindfulness-based third-wave CBT and forest bathing. Pilot results from this multi-level evaluation demonstrate that TIERRA holds promise as a sustainable, community-based intervention that promotes mental health and healing among Latine communities.

The primary objective of this paper was to evaluate the CBPR-based co-development and initial implementation of TIERRA via the PRISM and embedded RE-AIM framework. Results specifically demonstrate that a co-designed, nature-based intervention is both feasible and acceptable within the Latine community. Participant engagement (86% completion rate and 81% average session attendance) was high, particularly given the high attrition rates often observed in community mental health interventions (52, 53). Furthermore, the adoption of the task-shifting intervention by promotoras and the HLCC may reflect capacity building and the establishment of sustainable community infrastructure (34).

While preliminary effectiveness data showed significant reductions in stress (*p* = 0.026) and depression (*p* = 0.031), findings for anxiety and PTSD did not reach statistical significance. The non-significant trends may reflect a limited sample size, the need for more tailored measures to accurately capture experiences of cumulative trauma, the need for refining the intervention, or interference from other contextual factors, such as the dynamic sociopolitical climate. Specifically, the 2024 election occurred during the intervention timespan, with multiple participants alluding to its impact on their well-being. The most robust outcomes include increases in sense of community (*p* < 0.001) and belongingness (*p* = 0.009). These results highlight radical hope and collective resilience, both critical for navigating the systemic and environmental stressors unique to this community context. Findings about the effectiveness of the intervention will be discussed at greater length in a subsequent paper.

### 4.1 Culturally responsive development and adaptation

The intervention sought to provide culturally tailored healing in a non-traditional and relevant community context. An example of tailoring includes initiating the intervention in Spanish prior to developing an English adaptation. Adaptations emphasized cultural congruence, participant agency, and trauma-informed practice. Mindfulness and forest bathing were introduced through a decentered and integrative practice that acknowledged their roots in Eastern philosophy and their spiritual underpinnings (54). This aligned well with the Latine cultural value of esperanza (8). The adaptation of mindfulness for the Latine community also represents the Buddhist doctrine of equitable practice, echoing values central to the intervention (22, 54).

Content-specific adaptations to culture included discussions of *machismo*, relational forest bathing exercises, and contextualizing dialectical behavior therapy (DBT) skills; they exemplify community-relevant tailoring. The curriculum aimed to increase self-awareness of the impact of systemic and daily stressors for participants. DBT coping skills for these stressors were offered as flexible, non-judgmental invitations, as opposed to prescriptive solutions, empowering participants to tend to their own well-being without feeling invalidated. Similarly, interpersonal effectiveness and relational forest bathing exercises served to cultivate a balance between individuality and interconnected relationships, equipping participants to communicate their needs and engage in self-advocacy while recognizing their impact on other beings. Moreover, explicit discussions on *machismo* served a deeply validating function; by naming and acknowledging the impact of these patriarchal norms, participants could externalize this weight and choose with intention how they wish to navigate and respond in the future.

Anchored by the HEART and radical hope frameworks (8, 19), the intervention shifted focus from individual symptomatology to collective, multi-level healing. Nature was viewed as a sanctuary space for healing, consistent with the HEART framework’s promotion of collective expression and connectedness. Moreover, each session of TIERRA built toward the psychological practice of radical hope.

### 4.2 Reducing inequity through CBPR practices

Using a CBPR-informed approach to pilot implementation of TIERRA, we addressed structural barriers that have historically prevented Latine communities from accessing needed services. Access barriers, such as language difficulties, were circumvented by offering the sessions entirely in Spanish. Furthermore, spatial and structural obstacles within the external environment were circumvented by utilizing trusted, non-stigmatized community spaces like the library, and by providing on-site supervised childcare, and by coordinating transportation support; this reduced participant attrition. These efforts increased community trust, participation, and engagement with the intervention, and are reflected in participant engagement rates.

The promotora model further reflects an equitable practice that, when integrated within the CBPR principle of co-development, yielded a more contextually attuned, sustainable, and empowering intervention (55). Consistent with previous literature (56), TIERRA demonstrates that promotoras can serve as trusted cultural bridges who provide culturally competent care, thereby decreasing a major service gap. As embedded community members, their leadership ensured cultural resonance and built community capacity, establishing a foundation for long-term sustainability and community healing. Although CBPR is widely lauded in the literature for facilitating co-learning, sustainability, and equitable dissemination (11), true equitable power-sharing throughout the research process is often difficult in practice. TIERRA serves as a practical exemplar of adhering to CBPR principles, demonstrating how centering community voices can ensure that community members retain agency over their own healing processes (43, 44).

### 4.3 Application of implementation science frameworks

To ensure that the community-driven insights generated through CBPR were systematically reported, evaluated, and organized, the study relied on implementation science frameworks, which help mitigate the tension between clinical science and community practice (12). While CBPR drove intervention development, implementation science frameworks helped frame intervention evaluation. Specifically, the PRISM model allowed the research team to formally account for the impact of critical contextual determinants within the external environment (34). This included documenting efforts to navigate the accessibility of local terrain and safe green spaces, addressing the acute traumatic aftermath of the July 2022 mass shooting, and adapting to compounding chronic impacts of structural inequity. It also helped map multiple viewpoints during intervention development and while evaluating outcomes, which ensured a more community-centered approach that addressed specific barriers to care, such as conceptualizing supervised childcare to prevent participant attrition. Consistent with PRISM principles, there was adequate planning for the sustainability of the intervention from its initial conceptualization (i.e., through a promotora-led model and a train-the-trainer framework), providing necessary training support—including explicit modules on vicarious trauma to protect promotoras from burnout—and mechanisms for feedback that actively built local capacity. PRISM also helped account for the demographic, cultural, and contextual characteristics of the participants, promotoras, and management staff to ensure effectiveness at multiple levels. The use of CBPR with attention to and evaluation of contextual factors as suggested by the PRISM framework enhances the intervention’s real-world relevance (35).

The RE-AIM and PRISM frameworks provided a rigorous, structured methodology for contextually mapping interdependent implementation outcomes while centering sustainability (34). By systematically evaluating reach, effectiveness, adoption, implementation, and maintenance, the research team was able to empirically demonstrate the intervention’s value to community stakeholders, including participants, promotoras (staff), bilingual trainers (organizational leaders), and the organizations involved. Moreover, the framework proved complementary to CBPR. On the methodological front, combining CBPR with implementation science assisted in documenting processes and outcomes, which is often difficult for mixed methods CBPR interventions (57). Further, this resulted in initial success in reach, effectiveness, adoption, implementation, and maintenance. As further evidence of maintenance, the two community organization partners will continue offering TIERRA and related programming. This study serves as a reminder that to center health inequity, sustainability planning and capacity building should never be an afterthought, but rather a prime focus of intervention development.

### 4.4 Addressing gaps in the literature

Many evidence-based treatments for trauma and depression have been developed in controlled, academic settings with Western, educated, affluent participants, which has often led to high attrition rates and low cultural resonance when applied to marginalized individuals. TIERRA diverges from this standard paradigm. Although a pilot study, its impact on clinical symptoms is promising and warrants further exploration. It contributes to the growing body of literature on community health workers (56), demonstrating that when promotoras are equipped with tools and training, and are supported in their own healing journey, they can effectively provide mental health services for their community.

### 4.5 Limitations and future directions

There are several limitations to the study. First, a primary design limitation is the utilization of a pre- and post-test pilot design without a comparison group. Consequently, we cannot definitively rule out threats to internal validity, such as history or maturation (the natural passage of time), which may have also influenced participant outcomes (58). Second, the sample for the cultural adaptation of TIERRA was deeply embedded within the context and needs of the Latine community of Highwood, with a specific external environment—a suburban context with unique structural barriers. Consequently, the adapted protocol may not be immediately generalizable to Latine communities in more dense urban centers or more rural agricultural areas. Further, many participants had cultural ties to specific regions in Central America and given that cultural values may differ among Latine sub-groups (e.g., Central American vs. Caribbean), or from one generation to the next (immigrants vs. second or third generation), further adaptations are needed for other individuals from other Latine identities (59, 60). Future studies may also want to focus on Latine-identifying men to a greater extent. Adhering to a naturalistic design, the sample was a result of recruitment efforts driven by female-identifying promotoras; this may have resulted in a primarily Latine-identifying sample of women. The limited male participation may also have occurred because of differences in gender norms for Latine-identifying men, who may experience more cultural barriers such as stigma against seeking help (61, 62). Future iterations must therefore include targeted outreach through trusted males to actively increase male representation. Although the intervention was adapted, the outcome measures used to evaluate effectiveness were developed in Western contexts. Even with linguistic translation, these instruments may not possess full measurement equivalence of the construct (63, 64). Moreover, certain stressors and the impact of the intervention of cultural trauma were better captured qualitatively than quantitatively. Additionally, although the intervention was designed with sustainability in mind, longitudinal data have yet to be examined. Lastly, qualitative interviews only included a small percentage of the participants who may have been biased toward providing more positive feedback. Nevertheless, as a pilot intervention, our study provides considerations and methods that can lead to the co-development and successful implementation of interventions with, and for Latine-identifying individuals.

While the pilot data is promising, future research evaluating long-term contextual outcomes is needed. More research on the efficacy of intervention may also be warranted to rule out threats to internal validity and the impact of maturation. Future adaptations could also focus more concerted efforts to recruit Latine men. Future studies may also seek to adapt the intervention to other underserved community populations, with similar collective roots, in diverse geographic settings (i.e., in more urban or rural agricultural areas).

### 4.6 Clinical and community implications

This study offers several key implications for the fields of public health, clinical psychology, and community psychology. First, it suggests that scientific rigor and cultural congruence are not competing priorities. There are pathways for merging best practices; e.g., using implementation frameworks (PRISM and RE-AIM) alongside CBPR allows researchers to maintain rigor while centering marginalized voices. Second, its task-shifting and train-the-trainer model may add an example of a community healing intervention, urging intervention developers to consider non-traditional healers to promote community well-being. Thirdly, this work offers interdisciplinary insights for trauma-informed planning and spatial design by highlighting how accessible natural environments can serve as sanctuary spaces, i.e., functioning as an environmental pathway for post-traumatic community repair. Lastly, this study suggests that healing models that build community capacity and encourage empowerment can address structural racism.

## 5 Conclusion

Transforming Internal Experiences for Resilience and Restoration through Acceptance (TIERRA) demonstrates that addressing structural barriers and increasing inclusivity and cultural relevance can improve community well-being and increase nature access. Although often viewed as competing priorities, this study demonstrates that CBPR, PRISM, and RE-AIM can be effectively combined to achieve both scientific fidelity and cultural congruence, forging a path forward for more relevant, accessible, and effective care. TIERRA also provides a possibility for nature to be an equalizer––ensuring that healing through its restorative power becomes a possibility for many, as opposed to a privileged few.

### 5.1 Resource identification initiative

R Project (RRID: SCR_001905).

G*Power (RRID: SCR_013726).

## Data Availability

The raw data supporting the conclusions of this article will be made available by the authors, without undue reservation.
