# Supplementary material for: A multilevel evaluation of the development and implementation of a promotora-led nature-based intervention
Source: Front Public Health. 2026 Oct 27;14:1854138. doi: 10.3389/fpubh.2026.1854138 (PMC13752403; doi:10.3389/fpubh.2026.1854138)
Supplement: Supplementary file 1 [file Supplementary_file_1.docx]

**Table A1**

# *Session Content*

| **Session & Theme** | **Session Purpose** | **Core Activities and Purpose** |
| --- | --- | --- |
| **Session 1:** Overview and Creating Connections | The purpose of the first session is to introduce participants to the guiding framework and different concepts utilized in TIERRA. Most of the session focuses on psycho-education and rapport building. The session ends by planting the seed for Radical Hope | **Introduction:** Introduce facilitators and their roles; provide overview of the program's purpose  **Wishing Tree Exercise^c,d^:** Ask participants to share and rate on a scale of 0 (not painful) to 10 (extremely painful) *personal pain^c^* and a *collective community pain* they experience, and leave it on their *wishing tree^d^*    **Group Norms:** Establish agreements to build trust and safety    **Grounding^a,b^:** 5-4-3-2-1 grounding to introduce mindfulness and forest bathing.    **Sharing Circle^d^:** What trees, fruit, and flowers remind you of home?    **Closing Tea Circle^a^:** *Share one word or phrase that describes how you are feeling right now*    **At-Home Sit Spot Reflection^a,b^:** No sit spot for session 1 |
| **Session 2:** Mindfulness and Forest Bathing | To introduce participants to the practice of mindfulness and forest bathing. Participants will learn to cultivate the practice of presence in the here-and-now, which forms the basis of grounding, common to third-wave CBT and forest bathing. Participants will also be introduced to value-based living. | **Forest Bathing^a^:** Introduction to cultivating a practice for grounding; invitations: *Pleasures of presence, who or what is in motion, and cultivating a sit spot*    **Mindfulness^b^:** Discussing what mindfulness is and common misconceptions.    **WISE Mind^c,b, a^:** Moving away from being either too rational or too emotional to increase agency, intuition, and the heart sense    **Values^c^:** Introduction to how living based on values brings us closer to a meaningful life.    **Closing Tea Circle^a^**: *What would you like to say to feel complete today?*    **At-Home Sit Spot Reflection^a,b^:** To cultivate a spot to sit at outdoors for 10 minutes observing the outside world; prompt: *What are you noticing?* |
| **Session 3:** Acceptance and Radical Acceptance | To cultivate a deeper understanding of acceptance. Participants will explore willingness, acceptance, and Radical Acceptance. As we cannot hope for a better future without acknowledging our past and present, acceptance connects us to Radical Hope | **Forest Bathing^a^:** Invitation: *Of Life’s Journey and Letting go*  **Values^c,d,e^:** Discussing identified personal and collective cultural values. Prompt for the HEART framework    **Acceptance^a,b,c^:** Discussing the importance of acceptance versus avoidance with the help of the tug of war monster of stone in palm exercise    **Radical Acceptance^c^:** Encouraging the acceptance of difficult emotions without endorsing them or being complicit with the reason behind them.    **Closing Tea Circle^a^**: *In one word, you are invited to share something you accept.*    **At-Home Sit Spot Reflection^a,b^:** Upon returning to the same sit spot, reflect on *what it would be like to invite acceptance to sit with you?* |
| **Session 4:** Emotional Regulation | To learn to acknowledge and anticipate difficult emotions as a part of life, and cultivate coping strategies in advance. Participants will work towards gaining more agency over their emotions by regulating them. They will also learn more about how forest bathing and cultural practices can help with Emotional Regulation | **Forest Bathing^a^:** *Pleasures of Presence* with a stone  **Emotional Acceptance^b,c^:** Identifying emotions and exploring their function; Creating a greater capacity to hold pain and increasing long-term positive emotions.    **Reducing Emotional Vulnerability**^c^**:** Increasing agency by giving ourselves more choices.    **Cultural Reflection^c^:** Exploring the function of emotions to increase personal agency and choices.    **Closing Tea Circle^a^**: *Noticing how you are feeling right now, share where in your body you are feeling that emotion in this moment*    **At-Home Sit Spot Reflection^a,b^:** *How does your body meet the space? Notice the other nature beings that are also in this space with you. What would it be like to notice how you share the space together?* |
| **Session 5:** Distress Tolerance | To learn different techniques to manage difficult emotions quickly when we are in a state of heightened pain or distress. Participants will learn more about how forest bathing and cultural practices can help with distress tolerance. | **Forest Bathing^a^:** Worry stone exercise with *What’s in Motion*    **Distress Tolerance (DT) Skills^c^:** Teaching the difference between DT for emergency situations and emotional regulation as a long-term solution to stress.    **Cultural DT Strategies^c^:** Extending distress tolerance to Latinx cultural values and practices.  **Closing Tea Circle^a^**: *What are you noticing about yourself and your stone/nature being? What is your relationship like with your story now?*    **At-Home Sit Spot Reflection^a,b^:** *What would it be like to see that what is sometimes most simple is also most essential?* |
| **Session 6:** Interpersonal Effectiveness – Part I | The focus of the next two sessions is on fostering healthy relationships. The practices included can help us build connections, and help us think of ourselves in relation to other beings. This type of relational thinking forms the basis of collective thinking, which is one of the central tenants of Radical Hope | **Forest Bathing^a^:** Cultivating relationships with the group as well as nature; Invitation: Befriending a Tree    **Skills^c^:** Providing strategies for interpersonal effectiveness to reduce immense stress caused by relationships; Role Playing interpersonal skills to feel more connected to people we care about.  **Closing Tea Circle^a^**: *What are you noticing about your relationship to yourself, others, the earth, and/or your community?*    **At-Home Sit Spot Reflection^a,b^:** *What would it be like to share love with everything in the space for what it is?* |
| **Session 7:** Interpersonal Effectiveness – Part II |  | **Forest Bathing^a^:** Extending our experiences in nature to connect with others as a practice of deep listening and witnessing; Invitation: Gifts from the Forest  **Culture and Interpersonal Skills^c^:** Reflecting on culture and the different values and norms that influence our communication styles.    **Review^a,b,c,d^:** Re-visiting other learned skills    **Closing Tea Circle^a^**: *What would you like to say to feel complete today?*    **At-Home Sit Spot Reflection^a,b^:** W*hat would it be like to close your eyes and listen to the sounds of this place? What can you hear in this moment?* |
| **Session 8:** Radical Hope & Celebratory Potluck | To tie in everything learned so far, and utilize skills to cultivate the practice of Radical Hope | **Forest Bathing^a^:** A solo wander and collect a being to bring back and create a community art piece with the group    **Recap^a,b,c:^** Re-orienting to willingness^c^, radical acceptance^c^, and the need for effective communication.    **Radical Hope^a,b,c,d^:** Understanding the relationship between radical acceptance^c^, willingness^c^, forest bathing^a^, and radical hope^d^ as a part of radical healing^d^.  **Cultural Reflection^d^:** Celebrating cultural strengths, acknowledging past difficulties, making meaning, and having belief in a better future.    **Closing Tea Circle^a^** & Potluck: Re-visiting the wishing tree and celebrating with food prompts, blessings, and affirmations; prompt: *Where in your body do you notice Radical Hope?*    **At-Home Sit Spot Reflection^a,b^:** *What would it be like to create beauty, right here right now? Beauty that is meaningful to you in your life?* |

*Note.* This table consolidates the 8-week intervention curriculum. Core activities are bolded for structural clarity, while theoretical adaptations and reflections are italicized. DT = Distress Tolerance.

^a^ Denotes activities derived from forest bathing

^b^ Denotes activities derived from general mindfulness-based practices,

^c^ Denotes activities derived primarily from third-wave CBT; Namely, Dialectical  Behavior Therapy (CBT), or Acceptance Commitment Therapy (ACT)

^d^ Denotes activities comprising of components from the Radical Hope Framework
